# Glycoprotein 2 in health and disease: lifting the veil

**DOI:** 10.1186/s41021-021-00229-8

**Published:** 2021-12-03

**Authors:** Yingsong Lin, Masahiro Nakatochi, Naoki Sasahira, Makoto Ueno, Naoto Egawa, Yasushi Adachi, Shogo Kikuchi

**Affiliations:** 1 Department of Public Health, Aichi Medical University School of Medicine, 480-1195 Nagakute, Aichi Japan; 2 Division of Public Health Informatics, Department of Integrated Health Sciences, Nagoya University Graduate School of Medicine, 461-8673 Nagoya, Japan; 3 Department of Hepato-Biliary-Pancreatic Medicine, Cancer Institute Hospital of Japanese Foundation for Cancer Research, 135-8550 Tokyo, Japan; 4 Department of Gastroenterology, Hepatobiliary and Pancreatic Medical Oncology Division, Kanagawa Cancer Center, 241-8515 Yokohama, Japan; 5 Department of Internal Medicine, Tokyo Metropolitan Matsuzawa Hospital, 156- 0057 Tokyo, Japan; 6 Division of Gastroenterology, Department of Internal Medicine, Sapporo Shirakaba- dai Hospital, 062-0052 Sapporo, Japan

**Keywords:** GP2, Pancreas, Acinar cells, UMOD, Antibacterial, Genome-wide association study

## Abstract

In 2020, we discovered *glycoprotein 2* (*GP2*) variants associated with pancreatic cancer susceptibility in a genome-wide association study involving the Japanese population. Individuals carrying a missense coding variant (rs78193826) in the *GP2* gene resulting in a p.V432M substitution had an approximately 1.5-fold higher risk of developing pancreatic cancer than those without this variant. GP2 is expressed on the inner surface of zymogen granules in pancreatic acinar cells, which are responsible for the sorting, storage and secretion of digestive enzymes. Upon neuronal, hormonal, or other stimulation, GP2 is cleaved from the membrane of zymogen granules and then secreted into the pancreatic duct and intestinal lumen. While the functions of GP2 remain poorly understood, emerging evidence suggests that it plays an antibacterial role in the gastrointestinal tract after being secreted from pancreatic acinar cells. Impaired GP2 functions may facilitate the adhesion of bacteria to the intestinal mucosa. In this review article, we summarize the role of *GP2* in health and disease, emphasizing its functions in the gastrointestinal tract, as well as genetic variations in the *GP2* gene and their associations with disease susceptibility. We hope that its robust genetic associations with pancreatic cancer, coupled with its emerging role in gastrointestinal mucosal immunity, will spur renewed research interest in *GP2*, which has been understudied over the past 30 years compared with its paralog *uromodulin (UMOD).*

## Background

Glycoprotein 2 (GP2) was isolated from granule membranes of the rat pancreas in 1990 [1]. With a molecular mass of approximately 80 kDa, GP2 is expressed on the inner surface of zymogen granules of pancreatic acinar cells in various species [2]. Following the fusion of the membrane of zymogen granules (ZG) with the apical plasma membrane of pancreas acinar cells that is triggered by neuronal, hormonal, or other stimulation, GP2 is cleaved from the membrane of ZG and then secreted into the pancreatic duct and intestinal lumen [3]. Although GP2 was initially found to be expressed almost exclusively in the pancreas, its roles in the pathogenesis of pancreatic diseases are largely unknown. Perhaps coincidently, in 2020, we discovered *GP2* variants associated with pancreatic cancer susceptibility in a genome-wide association study (GWAS) involving the Japanese population [4].

Based on a ‘hypothesis-free’ approach, GWASs have revealed numerous disease-associated variants that stand the test of time [5]. Most importantly, GWASs offer fresh insights into the biological bases of complex diseases; one representative example is the unexpected revelation of complement system involvement in the pathogenesis of age-related macular degeneration [6]. For pancreatic cancer, at least 23 susceptibility loci have been identified by GWASs involving individuals of European descent [7]. However, whether these loci also exist in non-European populations remains unknown, as minor allele frequencies and linkage disequilibrium patterns differ across populations. Therefore, we conducted a meta-analysis of three GWASs with the largest sample sizes in East Asian populations. In addition to replicating the majority of the GWAS loci reported in European populations, we also identified robust, relatively large effect-size associations (odds ratio=1.46, a larger effect size than other variants reported in previous pancreatic cancer GWASs) of a coding missense variant (rs78193826: C>T; p.V432M) in the *GP2* gene with pancreatic cancer [4].

Stumbling upon *GP2* is only the beginning. Following the serendipitous association of this GWAS “hit” with pancreatic cancer, we aimed to elucidate its functions, with clinical application being the final goal. In this review article, we summarize the role of *GP2* in health and disease, with an emphasis on its functions in the gastrointestinal tract, as well as genetic variations in the *GP2* gene and their associations with disease susceptibility.

### Key discoveries about GP2

**Fig. 1:**
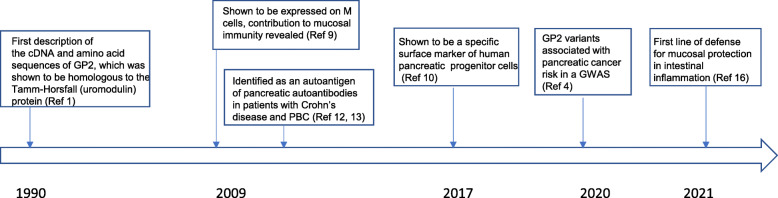
Timeline of key discoveries related to GP2

The key discoveries regarding GP2 are summarized in Fig. 1. In the early 1990 s, Fukuoka et al. made several prominent discoveries about *GP2*, including its expression on pancreatic acinar cells, its sequence similarity with *UMOD* (a flanking gene encoding uromodulin), and its classification as a glycosylphosphatidylinositol (GPI)-anchored protein, among others [1, 3]. The finding regarding the exclusive expression of GP2 in pancreatic acinar cells drew wide attention in the field, prompting ensuing research efforts to elucidate its biological functions. Given that GP2 is the major membrane protein of zymogen granules in pancreatic acinar cells, alterations in the *GP2* gene may affect the storage, sorting, and secretion of digestive enzymes. However, to the surprise of researchers, abrogating these presumable functions of *GP2* in knockout mice did not induce changes in either the morphology or functions of the pancreatic exocrine system [8]. Indeed, the roles of *GP2* remained enigmatic until 2009, when a Japanese group led by Hiroshi Ohno of RIKEN revealed its expression on mouse and human M cells in the small intestine, where it bound to FimH-expressing Gram-negative bacteria, such as *Escherichia coli* (*E. coli*) and *Salmonella* [9]. Their elegant work illuminated the contribution of GP2 to the gut mucosal immune system as a bacterial uptake receptor. In 2017, Cogger et al. identified GP2 as a specific marker of human pancreatic progenitors [10] Furthermore, on the basis of isolated GP2+ human pancreatic progenitors, Ameri et al. generated glucose-responsive beta cells that could be used in future diabetes cell therapies [11], making a case for the potential broad clinical application of GP2. Another important discovery related GP2 to inflammatory bowel diseases (IBDs), the incidence rates of which are increasing worldwide. GP2 was identified as an autoantigenic target in Crohn’s disease (CD) and primary sclerosing cholangitis [12, 13]. Accordingly, mucosal loss of tolerance to GP2 might be used as a biomarker to improve the diagnoses and prognoses of these diseases [13]. In 2020, we identified *GP2* variants associated with pancreatic cancer risk in a GWAS involving the Japanese population, providing further evidence that the leading missense variant in the *GP2* gene, rs78193826, is a functional, Asian-specific variant [4]. Despite these key findings, a systematic understanding of the biological and clinical value of GP2 is lacking. In particular, the biological functions of GP2 in the development of pancreatic diseases, including pancreatitis and pancreatic cancer, remain poorly understood.

### GP2 expression and domain structure

**Fig. 2:**
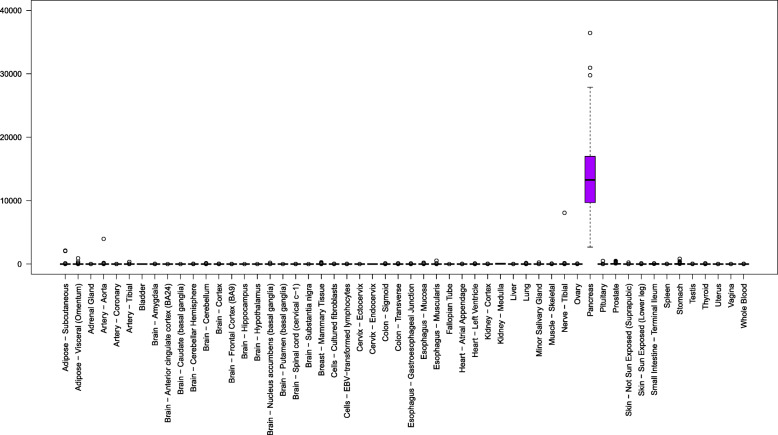
GP2 tissue expression according to the GTEx V8 (ENSG00000169347.16). GP2 is predominately expressed in the pancreas. The vertical axis indicates the TPM (transcripts per million) value, and the horizontal axis shows various tissues. Shown in the box plot are median, 25th, and 75th percentiles. The whiskers extend to the most extreme data point, which is no more than 1.5 times the interquartile range from the box

GP2 is predominately expressed in the pancreas (Fig. 2). Genotype-Tissue Expression (GTEx) RNA-seq data showed that the median expression level (TPM, 25th and 75th percentiles) was 15633.4 (11853.9 and 20143.9) among 248 samples [14]. Specifically, exocrine glandular cells exhibited the highest level of expression, followed by endocrine cells and mixed cell types [15], whereas ductal cells exhibited no or very low levels of expression. In addition to the pancreas, GP2 is also expressed in mouse and human M cells [9], and a recent study on the distributions of GP2 in the mouse digestive system revealed GP2 expression throughout the lumen of the small intestine and colon [16].

**Fig. 3:**
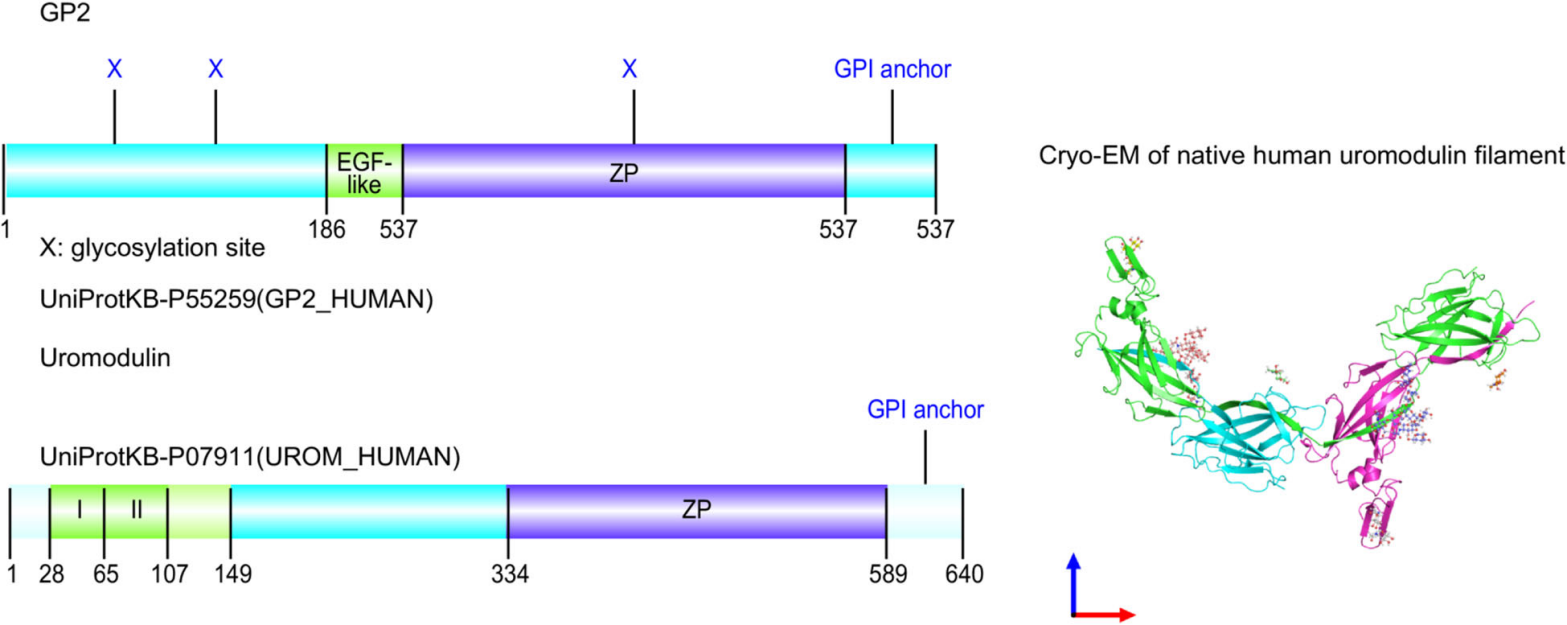
Domain structures of GP2 (upper left) and uromodulin (lower left). Both are GPI-anchored proteins featuring a ZP domain of ~260 amino acids. Cryo-electron microscopy (EM) of native human uromodulin filament is shown on the right (EMBO J e106807 https://www.ebi.ac.uk/pdbe/entry/pdb/6tqk)

High sequence similarity between rat GP2 and uromodulin was noted in an earlier study, in which these glycoproteins exhibited 86% similarity, with 53% identical C-terminal sequences [3]. Similarly, human GP2 and uromodulin sequences obtained from the UniProt databases were shown to be 41.6% identical, sharing 271 identical positions [17]. The molecular architecture of GP2 has not been firmly established, but it is generally accepted that GP2 comprises a cysteine-rich domain (D8C), an epidermal growth factor (EGF)-like domain, an asparagine-linked glycosylation site, and a zona pellucida (ZP) domain (Fig. 3A). Overall, the domain structure of GP2 is similar to that of uromodulin, which also features a ZP module (Fig. 3B). Both GP2 and uromodulin are attached to the membrane through a GPI anchor and are apically secreted into the extracellular compartment. Notably, the ZP domain, a conserved module of ~260 amino acids, is also found in an increasing number of other glycoproteins, including oocyte ZP proteins (ZP1, ZP2, ZP3), tectorins, transforming growth factor (TGF)-β receptor, and endoglin [18]. These glycoproteins are thought to have a similar function, as they all form filamentous homopolymers through their ZP domains [18]. Missense or frameshift mutations in the *ZP* genes can result in defective polymerization into filaments and have been linked to female infertility, hearing loss, and other human pathologies [19]. Based on both sequence and structural similarities, it is highly likely that *GP2* and *UMOD* are paralogs that derived from gene duplication—a major force driving evolution.

### Genetic variants in the ***GP2*** gene and their associations with diseases and traits

The *GP2* gene spans chromosome16:20,320,894-20,339,130 (Ensembl ID: NSG00000169347, hg19) and has 18,236 base pairs. According to gnomAD (v2.1.1), 137 synonymous, 274 missense, and 31 pLOF (predicted loss of function) variants have been identified in the *GP2* gene [20]. However, the clinical significance of these *GP2* variants remains to be determined, with only four single nucleotide variants (SNVs, rs76993218, rs115115341, rs141956527, and rs79104004) having been annotated in the ClinVar database [20]. As a result, none are predicted to be “pathogenic”.

**Fig. 4:**
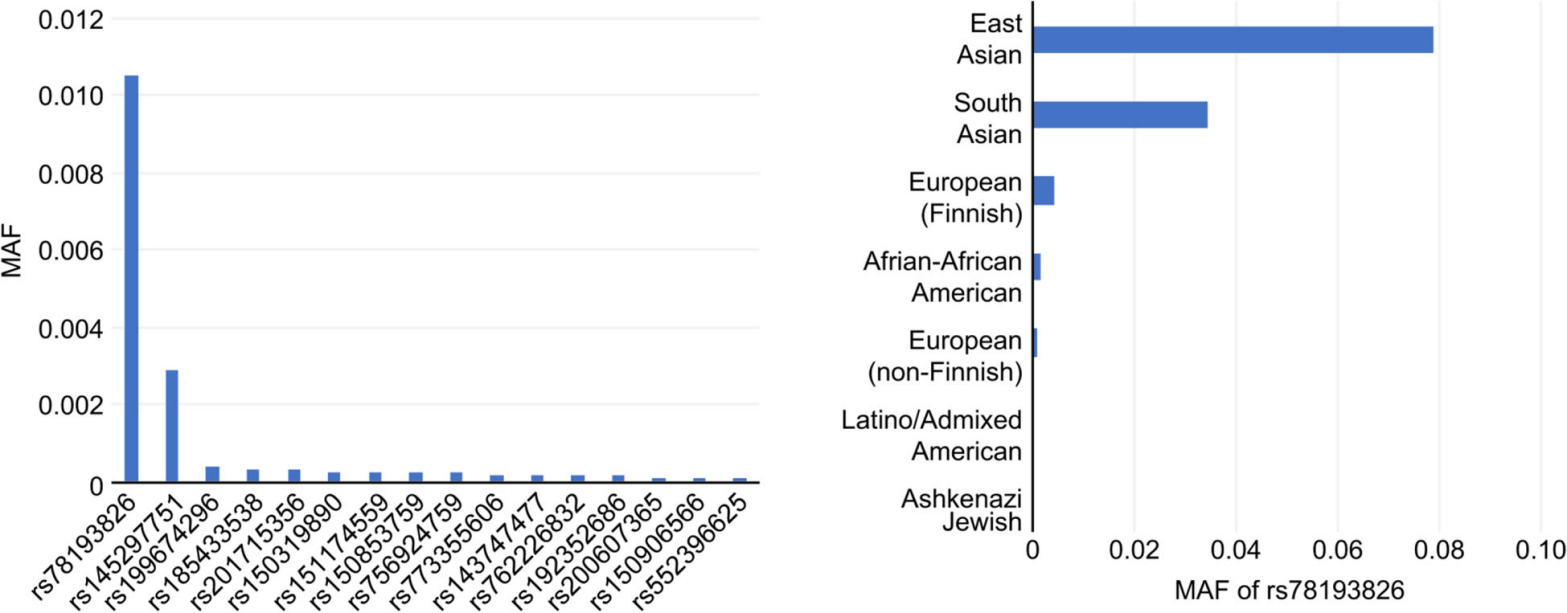
Missense variants in the GP2 gene sorted by MAF (left) and MAFs of the GP2 coding variant rs78193826 sorted by population (right)

As mentioned earlier, we identified the strongest GWAS “hit” for pancreatic cancer in the *GP2* gene, where a total of 10 variants were significantly (*p*<5 × 10^−8^) associated with the risk [4]. The lead variant is rs78193826, a missense variant (C>T: p.V432M) located within the coding region of *GP2*. Individuals carrying the risk allele T had an approximately 1.5-fold increased risk of pancreatic cancer compared with individuals with the alternative allele C. Of interest are wide variations in the risk allele frequency of rs78193826 across populations, as it ranges from 3 to 8% in Asian populations versus nearly 0% in populations of European and African ancestry. This apparent difference suggests that rs78193826 is a population-specific variant (Fig. 4). Moreover, genetic variations in this SNV are likely to be “deleterious” based on several bioinformatics tools, such as Sorting Intolerant from Tolerant (SIFT) [21] and Combined Annotation Dependent Depletion (CADD) [22]. CADD applies a framework that integrates multiple annotations into one metric based on contrasting variants that survive natural selection with simulated mutations [22]. Remarkably, rs78193826 has a CADD score of 19.8 (Hg38), the highest among all 274 missense variants in the *GP2* gene. One interesting question is why this allele was selected for and increased to a relatively high frequency in only Eastern Asian populations (Fig. 4), as opposed to being wiped out by natural selection over the course of evolution. Variants predicted to be deleterious by multiple algorithms are considered more likely to undergo intense selection purification [23]. Future evolutionary genomics analyses should further elucidate the selective pressure that might have shaped the *GP2* allele frequencies over time in different populations.

Another aspect of *GP2* variants is whether they are pleiotropic, defined as a single genetic variant associated with more than one phenotype. Previous GWASs have suggested widespread pleiotropic effects of SNVs across the genome [24]. Pleiotropic SNVs are more likely to be structurally functional and located in exonic regions than nonpleiotropic SNVs [24]. A search of the GWAS catalog revealed that *GP2* variants are associated with body mass index (BMI), type 1 and 2 diabetes, acute myeloid leukemia, and sleep quality in addition to pancreatic cancer [25–30]. Notably, the *GP2* variant rs117267808, which is in complete linkage disequilibrium (r^2^=1 according to 1000 genomes phase 3 JPT) with the *GP2* lead variant rs78193826, was associated with both pancreatic cancer and type 2 diabetes in the Japanese population [26]. The risk alleles for type 2 diabetes and pancreatic cancer were concordant at these two variants. Furthermore, the *GP2* variant rs1259579, located ~60 kb downstream of *GP2*, was one of the three novel variants associated with BMI in a GWAS meta-analysis involving East Asians [27]. These findings suggest that variants surrounding GP2 exert pleiotropic effects on multiple phenotypes. Further investigations into pleiotropy are warranted, as such knowledge may shed light on shared genetic mechanisms underlying the epidemiological associations between obesity/diabetes and pancreatic cancer risk.

### Biological functions of GP2

The role of GP2 in the pancreas and other organs remains unknown. Although this question has been addressed in a few studies over the past 30 years, the answer has not been completely elucidated. However, emerging evidence suggests that GP2 exerts antibacterial effects on the gastrointestinal tract after it is secreted from pancreatic acinar cells [16]. In fact, this antibacterial property parallels that of uromodulin in the urinary tract.

**Fig. 5:**
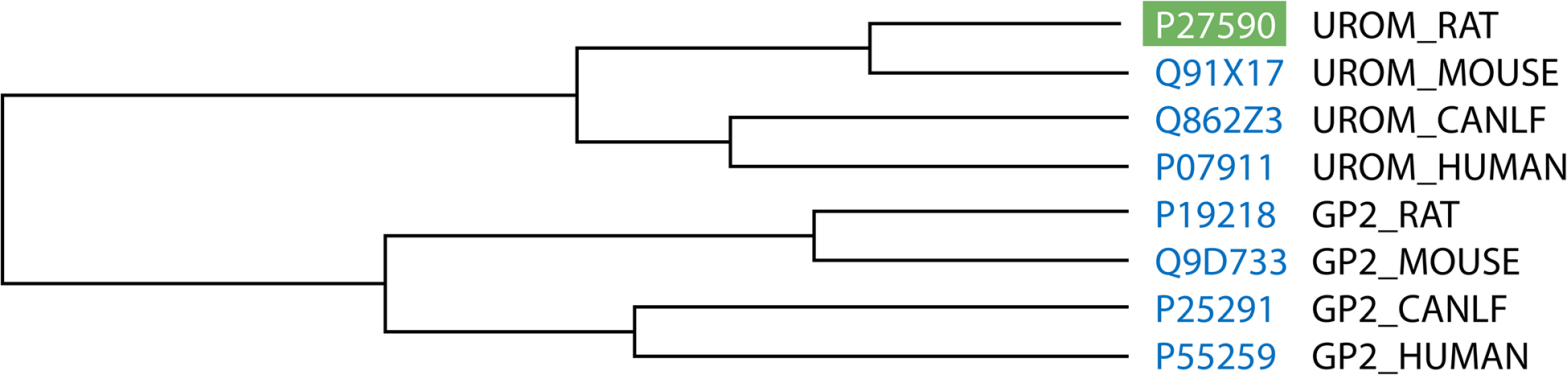
Evolutionary relationship between GP2 and UMOD. The phylogenetic tree was constructed using UniprotKB align

Given the structural similarities of GP2 and uromodulin, whether GP2 is functionally similar to uromodulin became a subject of interest shortly after its discovery. Unlike GP2, uromodulin, isolated by Tamm and Horsfall in 1952 (40 years earlier than *GP2*), has been extensively studied at both the structural and functional level [31, 32]. The domain structure of uromodulin has been well characterized, with a recent cryo-EM-based study succeeding in capturing the binding of uromodulin with *E. coli* in the urinary tract [32]. Uromodulin plays a pivotal role in the defense against urinary tract infections by forming filaments that prevent bacterial adhesion to glycoproteins of the urinary epithelium and promote pathogen clearance [31]. Furthermore, rare and common genetic alterations have been associated with a variety of disease outcomes, including those of chronic kidney diseases and hypertension [31]. Prospective cohort studies have demonstrated that urine uromodulin levels are associated with an increased risk of chronic kidney diseases [33]. On the other hand, GP2 has been understudied, with GP2 discoveries lagging behind those of uromodulin. The three-dimensional structure of the GP2 protein has not yet been constructed, and its biological functions in the pancreas and other organs remain poorly understood. However, the veil has been gradually lifted on GP2. To assess the hypothesis that GP2 and uromodulin share the ability to bind bacteria, Yu et al. performed an *in vitro* binding assay, showing that GP2 bound to *E. coli* expressing Type 1 fimbria [34]. Their findings indicated that the binding of GP2 to Type I fimbria may serve as a physical barrier and as a molecular decoy for bacterial adhesion, a function similar to uromodulin in the kidney. The seminal work by Hase et al. revealed the role of GP2 in the mucosal immune response to intestinal bacteria [9]. GP2 was shown to be expressed on M cells of the small intestine, where it served as a bacterial uptake receptor [9]. Building on the abovementioned work, Kurashima and colleagues further demonstrated that GP2 was widely distributed in the lumen of the digestive system and that its secretion from pancreatic acinar cells was increased upon the elevation of TNF-α levels during gut inflammation [16]. This latter finding suggests that intestinal GP2 controls bacterial invasion into intestinal epithelial cells, especially in an inflammatory environment. Collectively, the emerging evidence suggests that GP2 is a key factor in the intestine-pancreas axis, exerting antimicrobial effects in the gastrointestinal tract after being secreted from pancreatic acinar cells. Nevertheless, GP2 likely acquired not yet known novel functions after gene duplication because original and duplicate DNA sequences can acquire different mutations, resulting in new functions and differential patterns of expression [35]. Thus, elucidating the evolutionary relationship between GP2 and uromodulin could further our understanding of GP2 functions (Fig. 5).

### Clinical relevance

The clinical relevance of GP2 remains elusive despite being investigated in genetic, biomarker, and database studies. With the abundant expression of GP2 in pancreatic acinar cells, earlier studies focused on its role in pancreatic diseases. As GP2 is possibly involved in intraductal plug formation—an initial event of pancreatitis—after being secreted into the pancreatic duct, several previous studies attempted to identify both rare and common mutations in the *GP2* gene associated with chronic pancreatitis [36–38]. Three common polymorphisms in exons 3, 6, and 9 were identified in a candidate gene study involving 661 French patients with chronic pancreatitis [36]. Of them, the minor allele frequencies of two SNVs, c.348 C>T (rs12930599) and c.1275 A>C (rs1129818), differed significantly between idiopathic chronic pancreatitis (ICP) patients and control subjects, suggesting that these 2 synonymous SNPs are associated with the risk of ICP. Another functional study demonstrated that the SNP c.1275 A>C potentially influences the formation of truncated transcripts [34]. Furthermore, plasma GP2 levels were measured by the enzyme-linked immunosorbent assay (ELISA) in a clinical study involving patients with pancreatic diseases and control subjects [39]. The results indicated that GP2 was a better biomarker for acute pancreatitis than amylase, as it had higher sensitivity and specificity values.

In addition to pancreatic diseases, GP2 has been linked to multiple disease phenotypes, including CD, ulcerative colitis (UC), and primary sclerosing cholangitis [12, 13]. Notably, GP2 has been identified as the major autoantigenic target recognized by CD-specific pancreatic autoantibodies (PABs) [13]. A previous study reported that the prevalence of anti-GP2 PABs was 32% among patients with CD and 23% among patients with UC, whereas no anti-GP2 autoantibodies were detected in healthy control subjects [40]. These findings suggested the utility of PABs as a highly specific serologic marker for IBD. Decreased GP2 expression on the surface of microbial cells in CD patients may facilitate the adhesion of bacteria to the mucosa and promote inflammation. Interestingly, a recent GWAS revealed that common variants within the genomic regions surrounding the susceptibility loci for UC, CD, and chronic pancreatitis were associated with pancreatic cancer [41].

**Fig. 6:**
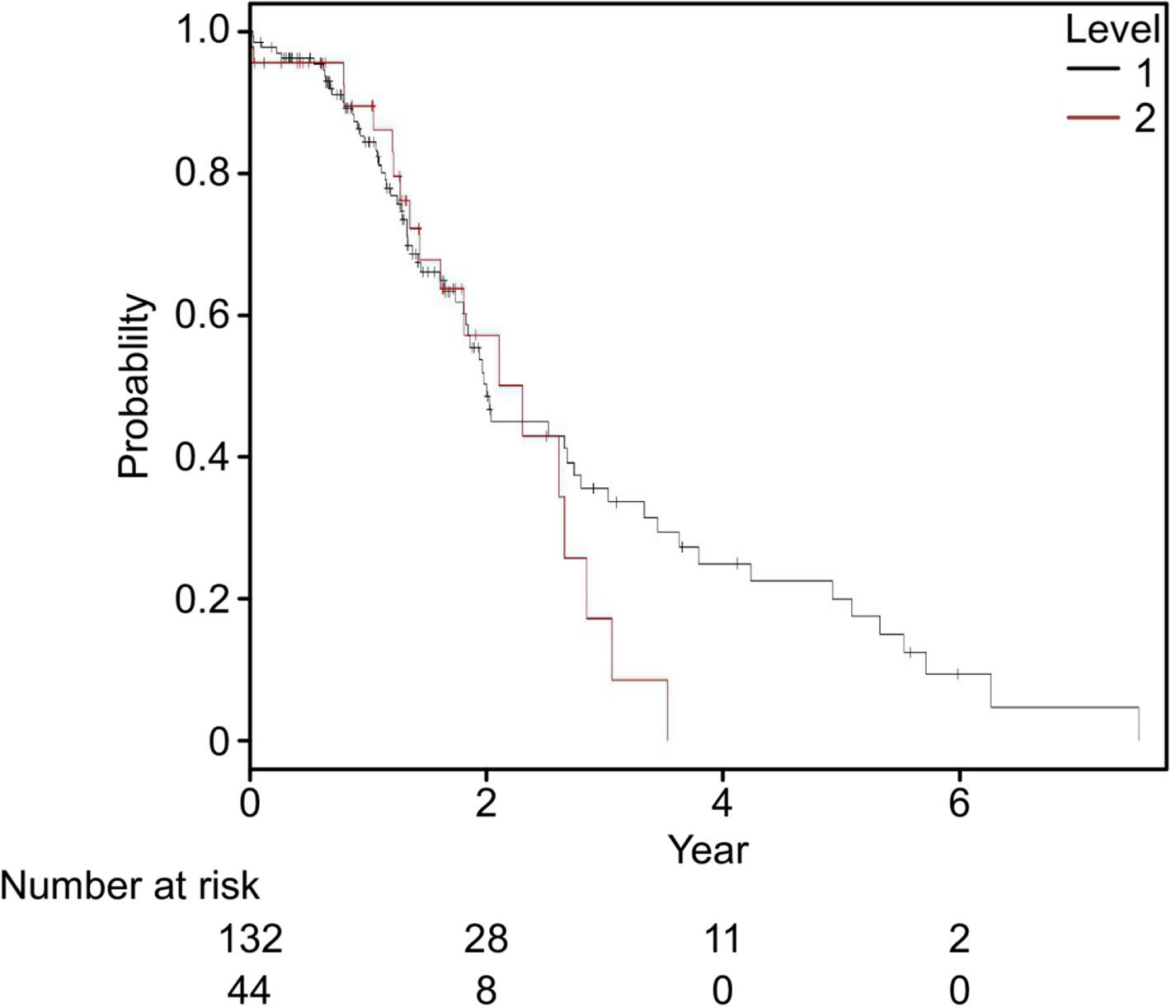
Survival analysis of 176 patients with pancreatic cancer patients based on GP2 mRNA expression (cutoff 128.5 FPKM)

A publicly available database provided a glimpse of the clinical relevance of GP2 as a prognostic marker. Survival analysis of 176 pancreatic cancer patients included in the TCGA database [42] showed no significant survival differences between the groups with high and low GP2 mRNA levels (log-rank P value=0.28) (Fig. 6). However, because the majority of the patients were diagnosed with stage II disease, the correlation between GP2 mRNA expression and the survival of patients in more advanced stages merits further investigation.

**Fig. 7:**
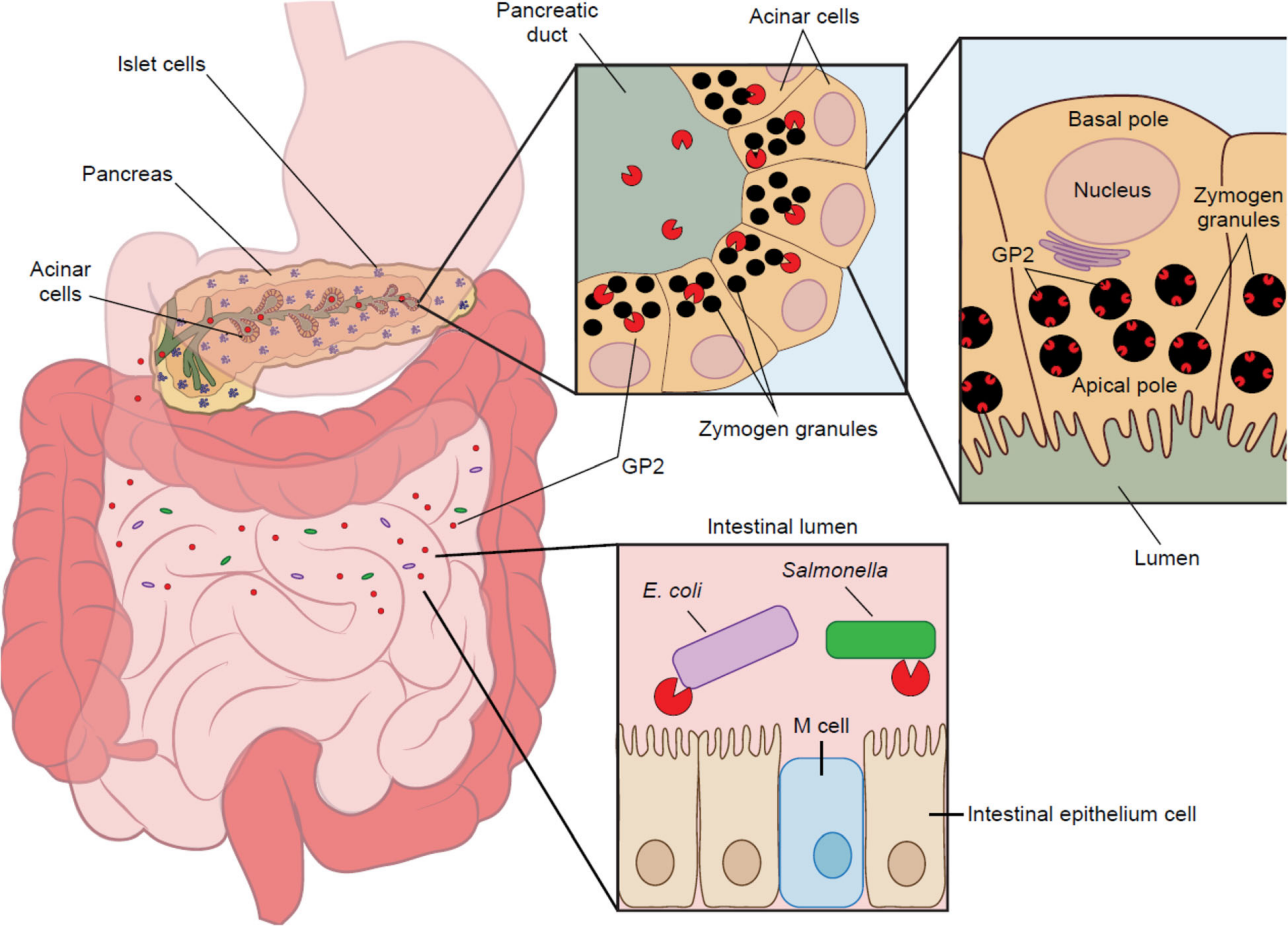
Functional roles of GP2. While the functions of GP2 remain poorly understood, emerging evidence suggests that it plays an antibacterial role in the gastrointestinal tract after being secreted from pancreatic acinar cells. Increasing evidence suggests that GP2 is a key factor in the intestine-pancreas axis, exerting antimicrobial effects in the gastrointestinal tract after being secreted from pancreatic acinar cells. In addition, previous studies have suggested that GP2 may be involved in the pathogenesis of pancreatitis, IBD (CD, UC), and pancreatic adenocarcinoma. Targeting *GP2* as well as other genes involved in innate immunity may offer insights into pancreatitis-pancreatic cancer association

Taken together, previous studies indicate that GP2 may have broad clinical relevance to gastrointestinal diseases (Fig. 7), but further studies are needed to establish its translational significance.

## Conclusions and perspective

As a highly conserved gene, GP2 is involved in pancreas development and associated with multitype disease phenotypes. Impaired GP2 functions may facilitate the adhesion of bacteria to the intestinal mucosa. In particular, emerging evidence suggests that *GP2* is a key factor in maintaining intestinal homeostasis by interacting with gut bacteria, a function similar to its paralog *UMOD*. Our GWAS revealed associations of *GP2* coding variants with the risk of pancreatic cancer, providing a promising target for functional follow-up. We hope that its robust genetic associations with pancreatic cancer, coupled with its emerging role in gastrointestinal mucosal immunity, will spur a surge of renewed research interest in *GP2*, a gene that has been understudied over the past 30 years compared with *UMOD*. Elucidating how inherited genetic variations in the *GP2* gene, both rare and common, alter the functions of the GP2 protein and further influence the susceptibility of pancreatic diseases could eventually help to identify drug targets. Targeting GP2 may also open up a new avenue for the detection and treatment of IBD, a morbidity that is common in Caucasians and is increasing in Asian populations [43].

## Data Availability

Not applicable.

## Abbreviations

GP2: glycoprotein 2
UMOD: uromodulin
GWAS: genome-wide association study
E. coli: Escherichia coli
GPI-anchored protein: glycosylphosphatidylinositol-anchored protein
IBD: inflammatory bowel diseases
CD: Crohn’s disease
GTEx: Genotype-Tissue Expression
EGF: Epidermal growth factor
ZP: zona pellucida
TGF-β: transforming growth factor (TGF)-β
SNV: single nucleotide variant
SIFT: Sorting Intolerant from Tolerant
CADD: Combined Annotation Dependent Depletion
BMI: body mass index
ELISA: enzyme-linked immunosorbent assay
UC: ulcerative colitis
PAB: pancreatic autoantibody
